# Salutary effects of glibenclamide during the chronic phase of murine experimental autoimmune encephalomyelitis

**DOI:** 10.1186/s12974-017-0953-z

**Published:** 2017-09-02

**Authors:** Volodymyr Gerzanich, Tapas K. Makar, Poornachander Reddy Guda, Min Seong Kwon, Jesse A. Stokum, Seung Kyoon Woo, Svetlana Ivanova, Alexander Ivanov, Rupal I. Mehta, Alexandra Brooke Morris, Joseph Bryan, Christopher T. Bever, J. Marc Simard

**Affiliations:** 1 Department of Neurosurgery, University of Maryland School of Medicine, 22 S. Greene St., Suite S12D, Baltimore, MD 21201-1595 USA; 2 Department of Neurology, University of Maryland School of Medicine, Baltimore, MD 21201 USA; 3 Department of Pathology, University of Maryland School of Medicine, Baltimore, MD 21201 USA; 4 Department of Physiology, University of Maryland School of Medicine, Baltimore, MD 21201 USA; 5 Research Service and MS Center of Excellence, Veterans Affairs Maryland Health Care System, Baltimore, MD 21201 USA; 6 Neurosurgical Service, Veterans Affairs Maryland Health Care System, Baltimore, MD 21201 USA; 7 Department of Pathology, University of Rochester Medical Center, Rochester, NY 14642 USA; 8 Pacific Northwest Diabetes Research Institute, 720 Broadway, Seattle, WA 98122 USA

**Keywords:** Multiple sclerosis, Experimental autoimmune encephalomyelitis, SUR1, TRPM4, SUR1-TRPM4 channel, *Abcc8*, Glibenclamide, Astrocyte

## Abstract

**Background:**

In multiple sclerosis (MS) and experimental autoimmune encephalomyelitis (EAE), inflammation is perpetuated by both infiltrating leukocytes and astrocytes. Recent work implicated SUR1-TRPM4 channels, expressed mostly by astrocytes, in murine EAE. We tested the hypothesis that pharmacological inhibition of SUR1 during the chronic phase of EAE would be beneficial.

**Methods:**

EAE was induced in mice using myelin oligodendrocyte glycoprotein (MOG) 35–55. Glibenclamide (10 μg/day) was administered beginning 12 or 24 days later. The effects of treatment were determined by clinical scoring and tissue examination. Drug within EAE lesions was identified using bodipy-glibenclamide. The role of SUR1-TRPM4 in primary astrocytes was characterized using patch clamp and qPCR. Demyelinating lesions from MS patients were studied by immunolabeling and immunoFRET.

**Results:**

Administering glibenclamide beginning 24 days after MOG_35–55_ immunization, well after clinical symptoms had plateaued, improved clinical scores, reduced myelin loss, inflammation (CD45, CD20, CD3, p65), and reactive astrocytosis, improved macrophage phenotype (CD163), and decreased expression of tumor necrosis factor (TNF), B-cell activating factor (BAFF), chemokine (C-C motif) ligand 2 (CCL2) and nitric oxide synthase 2 (NOS2) in lumbar spinal cord white matter. Glibenclamide accumulated within EAE lesions, and had no effect on leukocyte sequestration. In primary astrocyte cultures, activation by TNF plus IFNγ induced de novo expression of SUR1-TRPM4 channels and upregulated *Tnf*, *Baff*, *Ccl2*, and *Nos2* mRNA, with glibenclamide blockade of SUR1-TRPM4 reducing these mRNA increases. In demyelinating lesions from MS patients, astrocytes co-expressed SUR1-TRPM4 and BAFF, CCL2, and NOS2.

**Conclusions:**

SUR1-TRPM4 may be a druggable target for disease modification in MS.

## Background

In multiple sclerosis (MS) and experimental autoimmune encephalomyelitis (EAE), inflammation is perpetuated not only by infiltrating leukocytes but also by astrocytes [1–4]. Active white matter plaques are surrounded by reactive astrocytes that elaborate cytokines, chemokines, and neurotoxic factors that promote inflammation, act as leukocyte chemo-attractants, and directly injure tissues. Astrocytes may play similar pathogenic roles in subpial and gray matter demyelination. Of the currently approved disease-modifying therapies, most target the immune system exclusively, with an important exception being fingolimod (FTY720) [5, 6], a sphingosine-1-phosphate (S1P) receptor modulator that acts on S1P receptors on lymphocytes to cause their sequestration in lymph nodes, and acts on S1P receptors on astrocytes to reduce their inflammation-sustaining activities [7–10].

Sulfonylurea receptor 1 (SUR1)–transient receptor potential melastatin 4 (TRPM4) channels are not constitutively expressed under normal conditions, but are newly upregulated in a variety of central nervous system (CNS) disorders [11]. These channels are comprised of two subunits, a regulatory subunit, SUR1, and a pore-forming subunit, TRPM4, with co-assembly of the two required to form pathogenic channels [12]. Recent work showed de novo upregulation and pathological involvement of SUR1-TRPM4 in murine EAE [13]. In EAE, global gene silencing of either *Abcc8* or *Trpm4*, which encode SUR1 and TRPM4, respectively, as well as pharmacological inhibition of SUR1 by glibenclamide, were found to exert similar beneficial effects on inflammation, myelin preservation, and neurological function [13, 14], consistent with the requirement for functional SUR1-TRPM4 channels for disease progression. In those reports, treatment with glibenclamide was begun at the onset of clinical symptoms, 10 days after MOG_35–55_ immunization.

Our previous work showed that, during the chronic phase of EAE, SUR1-TRPM4 channels are expressed predominantly by reactive astrocytes in pathologically involved tissues that exhibit a significant inflammatory burden [13]. Based on this observation, we hypothesized that the channel would contribute to astrocyte-mediated inflammation in chronic EAE, and that treatment with glibenclamide during the chronic phase would be beneficial. Here, we tested this hypothesis by examining the effect of glibenclamide when administered beginning 24 days after MOG_35–55_ immunization, well after clinical symptoms of EAE had plateaued. In addition, we studied the role of SUR1-TRPM4 channels in cultured primary astrocytes activated by tumor necrosis factor (TNF) plus interferon γ (IFNγ), and we analyzed demyelinating lesions from MS patients to ascertain whether SUR1-TRPM4 channels are expressed by perilesional astrocytes in humans with MS.

## Methods

### Human tissues

Formalin-fixed brain tissues from MS patients were obtained from the Brain and Tissue Bank of the Rocky Mountain MS Center, Anschutz Medical Campus, University of Colorado Denver, Aurora, CO. Detailed pathological examinations of the human tissues were conducted at the University of Colorado Health Sciences Center, Department of Pathology. The tissues were further examined at the University of Maryland by a board certified neuropathologist (RIM). Tissue samples containing one or more demyelinating lesions were obtained from 9 patients, 6 females, aged 61 ± 2.6 years, and 3 males aged 60 ± 8.8 years. The post-mortem interval was 9.8 ± 1.4 h. Controls included 2 females and 4 males, aged 47 ± 9 years, who had died rapidly from non-neurological diseases (acute cardiovascular or respiratory disorders) [15].

### Murine EAE model

Female C57BL/6J mice were obtained from The Jackson Laboratory (Bar Harbor, ME). *Abcc8−/−* mice, obtained as described [13], exhibited neurological function and spinal cord histology indistinguishable from wild-type (WT) mice. Mice were housed under pathogen-free conditions in the animal facility of the University of Maryland, School of Medicine.

EAE was induced in female WT and *Abcc8*−/− mice at 8 weeks of age, as described [13]. EAE was induced with myelin oligodendrocyte glycoprotein 35–55 (MOG_35–55_) peptide (Biomer Technology, Pleasanton, CA) in complete Freund’s adjuvant containing *Mycobacterium tuberculosis* (H37RA; Sigma-Aldrich, St. Louis, MO). Mice were immunized by subcutaneous injection in the flank regions (left and right sides) with 200 μL total of an emulsion of MOG_35–55_ peptide (200 μg in 100 μL phosphate-buffered saline (PBS) plus 100 μL of complete Freund’s adjuvant containing 0.4 mg of heat-inactivated *M. tuberculosis*). Each mouse then received 400 ng of pertussis toxin (List Biological Laboratories, Campbell, CA) intraperitoneally (IP) on post-induction day (pid) 0 and pid-2.

*Abcc8*−/− mice with induced EAE were described previously [13], and so were used in the present study for only a single experiment that examined astrocyte expression of TNF, BAFF, CCL2, and NOS2 in EAE. WT mice with induced EAE were randomly assigned to receive no treatment or glibenclamide treatment beginning either on pid-12 or on pid-24 (these groups are referred to below as “EAE/glib-pid-12” and “EAE/glib-pid-24”). Treatment consisted of administering 10 μg glibenclamide IP daily to mice in the treatment groups until the end of the experiment (pid-40). A stock solution of glibenclamide was prepared by placing 25 mg glibenclamide (#G2539; meets USP testing; Sigma-Aldrich) into 10 mL dimethyl sulfoxide (DMSO). We diluted 200 μL of this solution into 9.8 mL PBS; mice received 200 μL of the final solution.

Scoring of disease severity was carried out as previously described [13]. From pid-1 onwards, mice were assessed daily for signs of paralysis by two independent observers blinded to treatment group. Mice were assigned a clinical score of increasing severity: 1, limp tail; 2, hind limb paresis; 3, complete hind limb paralysis; 4, hind limb paralysis and body/front limb paresis/paralysis; 5, moribund. End point evaluations included mean severity of disease over time and mean day of disease onset (first day of score > 0). Paralyzed mice (scores 3 and 4) were moved to individual cages where food and water were placed at cage floor level. The weight of EAE mice was measured every 2 days, and mice were euthanized if there was a loss of more than 20% in weight or if they become dehydrated.

On pid-40, mice were euthanized by IP injection of pentobarbital (> 100 mg/kg), followed by intracardial perfusion of 10% neutral-buffered formalin (15 mL; Sigma-Aldrich). The spinal cords were removed, immersion fixed for 24 h in formalin, and either cryoprotected 48 h in 30% sucrose prior to cryosectioning (7 μm) or they were paraffin embedded for sectioning.

### Histology, immunohistochemistry, and immunoFRET

*Mouse tissues.* For the analysis of tissues from pid-40 mice, paraffin or cryosections of the lumbar spinal cord were either stained with hematoxylin and eosin (H&E) or Luxol fast blue (LFB) or immunolabeled for either chromogen or fluorescence immunohistochemistry, as follows.

Chromogen immunohistochemistry was performed on paraffin sections, as described in [13], using VECTASTAIN Elite ABC Kits (#PK-6100; Vector Laboratories) and Mouse on Mouse (M.O.M.) Elite Peroxidase Kit (#PK-2200; Vector Laboratories). Primary antibodies were directed against: CNPase (1:1000; MAB326; EMD Milipore, Billerica, MA), PDGFRα (1:500; sc-338; Santa Cruz Biotechnology, Santa Cruz, CA), SMI-312 (1:1000; SMI-312R; Covance Inc., Gaithersburg, MD), cluster of differentiation (CD) 45 (1:1500; ab10558; Abcam, Cambridge, MA); CD20 (1:100; sc-7735; Santa Cruz Biotechnology); CD3 (1:200; ab5690; Abcam); p65 (1:200; GTX102090; Genetex, Irvine CA); CD86 (1:500; bs-1035R; Bioss,Woburn, MA); CD163 (1:500; bs-2527R; Bioss); TNF (1:500; sc-1350; Santa Cruz Biotechnology); BAFF (1:400; ab16081; Abcam); CCL2 (1:400; ab8101; Abcam); NOS2 (1:500; 482,728; EMD Millipore). Sections were counterstained with hematoxylin and were examined using bright-field microscopy. Quantification was performed by blinded observers using ImageJ software (National Institutes of Health, USA). Cell infiltrates (H&E) in white matter were quantified by counting the number of positive quadrants with inflammatory infiltrates and are reported as the percentage of the total number of quadrants examined. Immunolabeling experiments for specific markers were quantified based on the number of positive cells/field, with each field being a 435 × 325 μm rectangle, and with 7–9 fields covering the entire white matter of one coronal section of the lumbar spinal cord of each mouse.

Fluorescence immunohistochemistry was performed on cryosections. Myelin was assessed by immunolabeling for myelin basic protein (MBP) (1:500; Ab40390; Abcam) and by staining with LFB. Reactive astrocytosis was assessed by immunolabeling for GFAP (1:500; C9205; Sigma-Aldrich). Unbiased measurements of signal intensity within regions of interest (ROIs) were obtained using NIS-Elements AR software (Nikon Instruments, Melville, NY, USA). The area that was evaluated was the dorsal half of the white matter of one lumbar spinal cord section from each mouse. Specific labeling for MBP or LFB or GFAP within the ROI was defined as pixels with signal intensity > 1.5–2.0× background. Percent demyelination in EAE groups without and with treatment was computed based on measurements in normal (non-EAE) mice.

Double immunolabeling was performed for GFAP (1:500; C9205; Sigma-Aldrich) plus TNF (1:500; sc-1350; Santa Cruz Biotechnology) or BAFF (1:400; ab16081; Abcam) or CCL2 (1:400; ab7202; Abcam) or NOS2 (1:500; 482,728; EMD Millipore). Double immunolabeling also was performed for SUR1 (1:800; custom rabbit antibody) plus CD31 (1:200; 550,274; BD Biosciences; San Jose, CA). For visualization, we used fluorescent-labeled species-appropriate secondary antibodies [1:500, Alexa Fluor 488 (green) and Alexa Fluor 555 (red); Invitrogen/Molecular Probes, Eugene, OR] at room temperature. The specificity of the immunolabeling for all proteins was tested in control sections by incubation with pre-immune serum, or after pre-adsorption of the antibody with the peptides used as immunogens.

*Human tissues.* For the analysis of human tissues, cryosections (10 μm) were stained with H&E or LFB to identify appropriate areas for further evaluation by immunohistochemistry. We used immunolabeling for CD68 (1:200; ab955; Abcam), CD45 (1:200; ab10558; Abcam) and CD3 (1:200; ab5690; Abcam) to categorize demyelinating white matter lesions as either active, chronic active or chronic inactive, per De Groot et al. [16], as well as to analyze subpial/cortical demyelinating lesions. Tissues from the 9 MS patients yielded 13 white matter lesions, including 5 active lesions, 4 chronic active lesions, and 4 chronic inactive lesions. From these 9 cases, we also identified 6 demyelinating subpial/cortical lesions, each from a different patient. Preactive white matter lesions with “normal appearing white matter” were not included in this study.

Immunohistochemistry and immunoFRET were performed on cryosections using custom goat or custom rabbit anti-SUR1, custom chicken anti-TRPM4, or goat anti-TRPM4 (G-20, sc-27,540, Santa Cruz Biotechnology) antibodies, as described [12, 15, 17]. Slides were incubated with a mixture of 2% donkey serum (D9663; Sigma-Aldrich) and 0.2% Triton X-100 for 1 h at room temperature prior to immunolabeling.

Single-label immunohistochemistry was performed using biotin-conjugated secondary antibody. Sections were incubated for 30 min in PBS with 0.3% H_2_O_2_ to block endogenous peroxidase activity, after which sections were placed in the above blocking solution for 1 h. Following overnight incubation with anti-SUR1 antibody (1:800; custom rabbit antibody [12]) at 4 °C, sections were incubated with biotinylated secondary antibody (1:500; BA-1000; Vector Laboratories, Burlingame, CA) for 1 h. After washing in PBS, sections were incubated in avidin biotin solution (Vector Laboratories) and the color was developed in diaminobenzidine chromogen solution (0.02% diaminobenzidine in 0.175 M sodium acetate) activated with 0.01% hydrogen peroxide. The sections were rinsed, mounted, dehydrated, and cover-slipped with DPX mounting medium (Electron Microscopy Services, Hatfield, PA). Omission of primary antibody was used as a negative control.

Double-label immunohistochemistry was performed using fluorescent secondary antibodies. To identify cell-specific expression, double immunolabeling was performed for SUR1 (1:800; custom rabbit antibody) plus S100B (1:400; ab868; Abcam) or GFAP (1:500; C9205; Sigma-Aldrich) or CD68 (1:200; ab955; Abcam) or CD3 (1:200; ab5690; Abcam). To show co-localization, double immunolabeling was performed for SUR1 (1:800; custom rabbit antibody) plus TRPM4 (1:800; custom chicken antibody [12], as well as for SUR1 (1:800; custom rabbit antibody) plus BAFF (1:400; ab16081; Abcam), or CCL2 (1:400; ab7202; Abcam), or NOS2 (1:500; 482,728; Calbiochem, San Diego, CA). For visualization, we used fluorescent-labeled species-appropriate secondary antibodies [1:500, Alexa Fluor 488 (green) and Alexa Fluor 555 (red); Invitrogen/Molecular Probes, Eugene, OR] or, in the case of chicken anti-TRPM4 antibody, a FITC-conjugated secondary antibody (The Jackson Laboratory), all at room temperature. Sections were cover-slipped with polar mounting medium containing anti-fade reagent and 4′,6-diamidino-2-phenylindole (DAPI; Invitrogen). Immunolabeled sections were visualized using epifluorescence microscopy (Nikon Eclipse 90i; Nikon Instruments Inc., Melville, NY). Omission of primary antibody was used as a negative control. Specific labeling was defined as fluorescence intensity twice that of background.

Semi-quantitative analysis of SUR1 labeling intensity in various types of demyelinating lesions was performed as described previously for SUR1 [17]. Briefly, all sections had been immunolabeled as a single batch, and all images were collected using uniform parameters of magnification and exposure. Two areas encompassing demyelinating lesions were randomly selected for construction of a montage, with each montage composed of 4 images, each 300 × 400 μm, that were acquired at × 20 magnification. Images were evaluated independently by 2 observers for specific SUR1 immunoreactivity associated with each lesion type. Areas of maximum labeling were scored for each case using a semi-quantitative scale ranging from 0 to 4. The overall concordance between observers was more than 90%. In cases of disagreement, independent reevaluation was performed by a third observer to arrive at the final score. Co-localization of fluorescence signals in double immunolabeled sections (SUR1 plus BAFF or CCL2 or NOS2) was computed as Pearson’s correlation coefficient [18].

Antibody-based Förster resonance energy transfer (immunoFRET) for SUR1-TRPM4 was performed as described above [12, 17], except for the secondary antibodies (CY5-conjugated donkey anti-chicken and CY3-conjugated donkey anti-rabbit antibodies; The Jackson Laboratory), image acquisition using a Zeiss LSM710 confocal microscope, and controls that included omission of one or the other of the two primary antibodies.

### Fluorescence-activated cell sorting (FACS)

Normal (no EAE) mice were administered fingolimod (FTY720, SML0700, Sigma-Aldrich) (3 mg/kg once daily × 3 days by gavage) or glibenclamide (#G2539; Sigma-Aldrich; 10 μg/mouse once daily × 3 days IP). Untreated normal mice served as controls.

To evaluate circulating leukocytes, whole blood was collected aseptically by cardiac puncture using sterile K3 EDTA VACUTAINER blood collection tubes. The fluorochrome-conjugated monoclonal antibodies, FITC rat anti-mouse CD45 (553080), PerCP rat anti-mouse CD4 (553052), and PE rat anti-mouse CD8a (553032), 2 μg each, all from BD Biosciences, were added to 100 μL of whole blood in a 12 × 75-mm-capped polystyrene test tubes (2058; BD Biosciences). The blood was vortexed gently and was incubated for 30 min in the dark at room temperature, after which 2 mL of × 1 FACS Lysing Solution (349,202; BD Biosciences; × 10 diluted to × 1 in distilled water before use) was added. The mixture was vortexed gently and was incubated for 10 min in the dark at room temperature. The suspension was centrifuged at 500 × *g* for 5 min, the supernatant was removed, 2–3 mL of wash buffer (PBS with 0.1% sodium azide) was added, and the suspension was centrifuged at 500 × *g* for 5 min. The supernatant was removed and 0.5 mL of 1% paraformaldehyde solution (prepared in PBS with 0.1% sodium azide) was added to fix the cells. Counts of total CD45+ leukocytes, and CD45 + CD4+ and CD45 + CD8+ T-lymphocytes in the three treatment groups were determined by FACS (BD LSR II Flow Cytometer; University of Maryland, Center for Innovative Biomedical Resources) [19].

To evaluate the splenocytes, spleens were removed immediately after euthanasia and were placed in PBS on ice. They were then sliced into small pieces (∼1 mm^3^) in a Petri dish using surgical blades, and a single cell suspension was prepared by gently forcing the pieces through a 100-μm cell strainer. The resulting cell suspension was centrifuged and resuspended in red blood corpuscle lysis buffer (× 1 FACS Lysing Solution, BD Biosciences), was shaken at room temperature for 10 min, and was washed 2× with PBS with 0.1% sodium azide by centrifugation at 500 × *g* and resuspension at 4 °C. Isolated splenocytes were counted to estimate total cell numbers. Prior to incubation with antibodies, cells were fixed for 20 min in 1% formaldehyde solution (prepared in PBS with 0.1% sodium azide). Surface labeling was performed using the fluorochrome-conjugated monoclonal antibodies: PE hamster anti-mouse CD3e (#561824), APC rat anti-mouse CD4 (#553051), and PE-CY5 rat anti-mouse CD8a (#553034), all from BD Biosciences and was carried out in FACS buffer (BD Biosciences) with 1-h incubation at room temperature. Counts of total CD3+, CD3 + CD4+, and CD3 + CD8+ T-lymphocytes in the three treatment groups were determined by FACS (BD FACSCanto II system; University of Maryland, Center for Innovative Biomedical Resources).

### Glibenclamide accumulation in EAE lesions

Mice with symptomatic EAE (*n* = 3) and control (non-EAE) mice (*n* = 3) were studied. Bodipy-glibenclamide (TRITC (red); Thermo Fisher Scientific, Waltham, MA), 30 μg, was dissolved in 30 μL DMSO, then was added to 300 μL normal saline (NS). This solution was infused into the jugular vein of the anesthetized mouse, was allowed to circulate 1 h, after which the mouse was euthanized and perfused with NS to remove any intravascular signal. Cryosections of the lumbar spinal cord were either stained with H&E or were immunolabeled for fluorescence immunohistochemistry, as above. Immunolabeling of adjacent sections was performed for MBP (1:500; ab40390; Abcam) and albumin (1:100; ab106582; Abcam), with species-appropriate Alexa Fluor 488-conjugated secondary antibody (green).

### Astrocyte primary cultures

Astrocyte primary cultures were prepared from post-natal day 3–5 C57B6 mice or Wistar rats using a modified version of the method described [20]. Animals were euthanized with a barbiturate overdose, whereupon brains were dissected from the skull and were placed in ice-cold Dulbecco’s modified Eagle’s medium containing 4.5 g/L glucose (Invitrogen), supplemented with 10% fetal bovine serum, 100 units/mL penicillin, and 100 μg/mL streptomycin. The meninges were completely removed, and the cortices were isolated. Cortices were passed through 70- and 40-μm nylon meshes, and cell suspensions were plated in the above media. Astrocyte cultures were maintained to confluency for ~12 days, with media changes every 3 days. Confluent cultures were rotary shaken at 250 rpm for 18 h to detach contaminating cells, and were washed × 3 with the above media. GFAP immunolabeling demonstrated that cultures were > 95% GFAP+. Astrocyte cultures were maintained for experiments < 2 months.

To model the astrocyte response in MS/EAE, astrocyte cultures were exposed to TNF (20 ng/mL; 8902SC, Cell Signaling, Danvers, MA) plus IFNγ (20 ng/mL; 8901SC, Cell Signaling) overnight [1]. After activation, cells were studied by patch clamp electrophysiology to detect functional SUR1-TRPM4 channels, or they were processed to measure mRNA, as described below.

#### Patch clamp electrophysiology

Primary astrocytes, with or without TNF + IFNγ activation, were studied using patch clamp electrophysiology, as described [12, 21, 22]. Whole cell recordings were performed using a nystatin perforated patch technique to minimize disturbance of the intracellular milieu that causes rapid rundown of TRPM4 currents. Nystatin, 50 mg (Calbiochem) was dissolved in DMSO, 1 mL. Working solutions were made before the experiment by adding 16.5 μL nystatin stock solution to 5 mL of the base pipette solution to yield a final concentration of nystatin of 165 μg/mL and DMSO 3.3 μL/mL. To record whole cell macroscopic currents exclusive of K^+^ channels, such as K_ATP_, the extracellular solution contained (mM): CsCl 145, CaCl_2_ 1, MgCl_2_ 1, HEPES 32.5, glucose 12.5, pH 7.4; and the pipette solution contained (mM): CsCl 145, MgCl_2_ 8, and HEPES 10, and nystatin, 165 μg/mL, pH 7.2. The following parameters were used to elicit membrane currents: holding potential, − 50 mV; ramp pulses were from − 100 to + 10 mV, 4 mV/ms, applied every 15 s.

#### RNA isolation and quantitative real-time polymerase chain reaction

Mouse primary astrocytes, with or without TNF + IFNγ activation, were homogenized in Trizol Reagent (Thermo Fisher Scientific) and total RNA was isolated with Direct-zol™ RNA MiniPrep Kit (Zymo Research; Irvine, CA). To avoid contamination by genomic DNA, RNA was further purified with Amplification Grade DNase I (Invitrogen). The concentration of total RNA was determined by measuring the optical density at 260 and 280 nm. cDNA was synthesized from 1 μg of total RNA of each sample using SuperScript III Reverse Transcriptase (RT) Supermix (Thermo Fisher Scientific). The abundance of the various mRNAs in the samples was determined by quantitative polymerase chain reaction (qPCR) (ABI PRISM 7300; Applied Biosystems, Carlsbad, CA). qPCR reactions (25 μL) consisted of 1-μL cDNA template, Platinum SYBR Green SuperMix-UDG with ROX (Thermo Fisher Scientific), and gene specific primers. Reactions were incubated at 50 °C for 2 min and 95 °C for 2 min, were followed by 40 cycles of 95 °C for 15 s and 60 °C for 30 s, and were followed by melting curve analysis. No-template and no-RT reactions were used as negative controls in every experiment. Melting curve analysis was used to confirm the validity of experimental results. The primers used in this study are listed in Table 1. Data are reported as fold-change.

**Table 1:** Primer sequences used for qPCR

Gene	RefSeq	Primer sequence (5′ to 3′)	
		Forward	Reverse
*Abcc8*	NM_011510.3	GCCAGCTCTTTGAGCATTGG	AGGCCCTGAGACGGTTCTG
*Trpm4*	NM_175130.4	TGTTGCTCAACCTGCTCATC	GCTGTGCCTTCCAGTAGAGG
*Tnf*	NM_013693	GATCGGTCCCCAAAGGGATG	AGATGATCTGAGTGTGAGGGT
*Baff*	NM_033622	CTACCGAGGTTCAGCAACACCA	GAAAGCGCGTCTGTTCCTGTGG
*Ccl2*	NM_011333	GCTACAAGAGGATCACCAGCAG	GTCTGGACCCATTCCTTCTTGG
*Nos2*	NM_010927	TGGAGCGAGTTGTGGATTGTC	GGGCAGCCTCTTGTCTTTGA
*Rps18*	NM_011296	CGGAAAATAGCCTTCGCCATCAC	ATCACTCGCTCCACCTCATCCT

### Statistical analysis

Data are shown as mean ± standard error of the mean (SE). Non-parametric data (murine clinical scores and SUR1-immunolabeling scores) were analyzed using the Mann-Whitney or the Kruskal-Wallis test with Dunn’s post hoc comparisons, as appropriate. Parametric data were analyzed using the *t* test or the ANOVA with Fisher’s post hoc comparisons, as appropriate. Statistical analyses were performed using OriginPro version 8 (OriginLab Corp, Northampton, MA). For all experiments, *P* < 0.05 was considered to be statistically significant.

## Results

### Chronic phase glibenclamide improves neurological symptoms

**Fig. 1:**
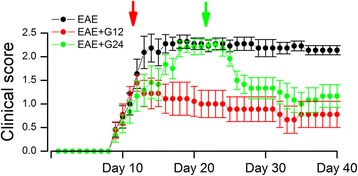
Chronic phase glibenclamide improves neurological symptoms in EAE. Mean ± SE of clinical scores following EAE induction, for untreated EAE mice, for EAE mice treated with glibenclamide starting on pid-12 (red arrow, red symbols), and for EAE mice treated with glibenclamide starting on pid-24 (green arrow; green symbols); 10–12 mice/group; see Table 2 for statistical analysis

Clinical scoring was assessed daily on pid-1–40. Untreated EAE mice showed a typical course of EAE, marked by worsening paralysis scores beginning on pid-9–12, peaking several days later, then persisting unchanged through to pid-40 (Fig. 1). The mean clinical score during the late disease period (pid-40) was 2.14 ± 0.10. The day of disease onset and the mean clinical scores at different times during the disease course are shown in Table 2.

**Table 2:** Summary of EAE clinical scores

Group	Incidence/total (%)	Number of mice dead	Disease onset (days)^a^	Clinical scores (mean ± SE)	
				pid-20^b^	pid-40^c^
EAE	11/11 (100%)	0	10.9 ± 0.71	2.27 ± 0.12	2.14 ± 0.10
EAE/glib-pid-12	10/10 (100%)	1	10.7 ± 0.27	1.20 ± 0.32	0.78 ± 0.28
EAE/glib-pid-24	12/12 (100%)	0	11.7 ± 0.80	2.21 ± 0.17	1.17 ± 0.24

^a^Disease onset is defined as the first day of a clinical score of one or more. There was no statistically significant difference in day of disease onset between groups

^b^The Kruskall-Wallis test indicated a significant difference between groups (*P* = 0.0257) on pid-20, with Dunn’s post hoc comparisons indicating that the EAE group was statistically different compared to the EAE/glib-pid-12 group (*P* < 0.05); the EAE and EAE/glib-pid-24 groups were not statistically different

^c^The Kruskall-Wallis test indicated a significant difference between groups (*P* = 0.0010) on pid-40, with Dunn’s post hoc comparisons indicating that the EAE group was statistically different compared to both the EAE/glib-pid-12 (*P* < 0.01) and the EAE/glib-pid-24 (*P* < 0.05) groups; the EAE/glib-pid-12 and EAE/glib-pid-24 groups were not statistically different

In murine EAE, pharmacological inhibition of SUR1 by glibenclamide beginning on pid-10 was found to yield improved clinical scores compared to untreated mice [13]. Here, we examined the effects of glibenclamide administered starting during the chronic phase of EAE. Mice were treated once daily with glibenclamide, 10 μg IP, beginning at pid-24 or, for comparison, at pid-12 and continuing through to pid-40. The clinical course of EAE/glib-pid-12 mice resembled that reported for mice beginning treatment on pid-10: progression of clinical symptoms was halted and clinical scores thereafter declined slightly then stabilized (Fig. 1). In the EAE/glib-pid-24 mice, clinical scores before treatment were as severe as in untreated mice (Fig. 1; Table 2). However, clinical scores improved noticeably within 2 days of beginning treatment and, by 1 week, scores had improved to levels similar to those in the EAE/glib-pid-12 group. The decrease in clinical severity with glibenclamide in the EAE/glib-pid-24 group persisted through the remainder of the experiment. In the EAE/glib-pid-24 group, the mean clinical score for the late disease period (pid-40) was 1.17 ± 0.24, which was significantly different from that in untreated EAE mice (2.14 ± 0.10), but not statistically different from the scores in the EAE/glib-pid-12 group (0.78 ± 0.28) (Fig. 1; Table 2).

### Chronic phase glibenclamide reduces demyelination and axonal loss

**Fig. 2:**
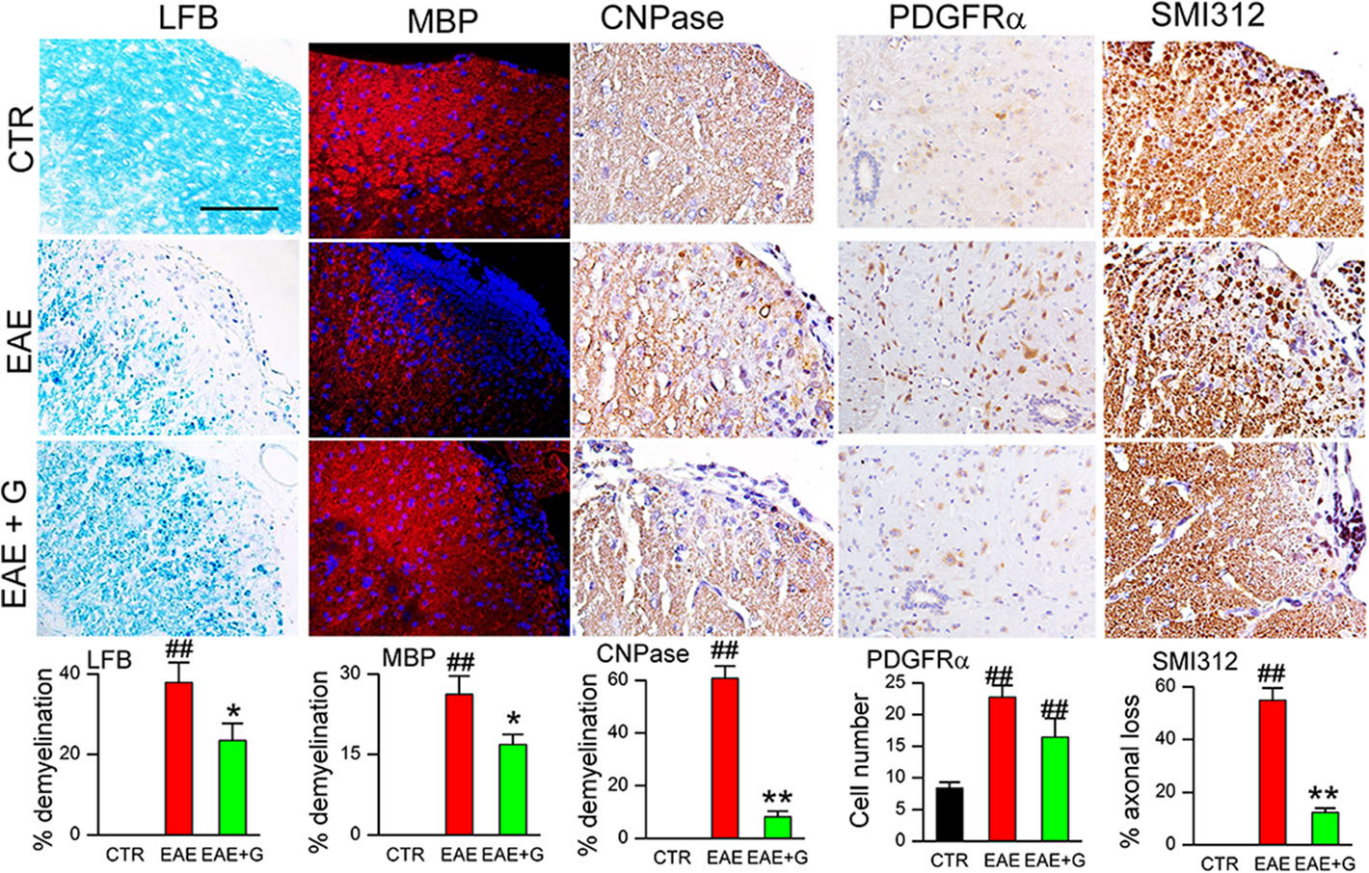
Chronic phase glibenclamide reduces demyelination in EAE. White matter of lumbar spinal cord sections from non-EAE control (CTR), untreated EAE mouse (EAE), and EAE mouse treated with glibenclamide starting on pid-24 (EAE + G), stained with Luxol fast blue (LFB) (myelin) or immunolabeled for myelin basic protein (MBP) (myelin), CNPase (mature oligodendrocytes), PDGFR-α (oligodendrocyte progenitor cells; OPC) or SMI-312 (neurofilament marker), as indicated; nuclei stained with DAPI in the MBP sections; original magnification, × 200 (LFB) or × 400 (all immunolabelings); *bar graphs*: percent of quadrants with myelin loss by LFB staining, MBP immunolabeling, or CNPase immunolabeling; quantification of PDGFR-α-expressing OPC near the central canal; percent of quadrants with axonal loss by SMI-132 labeling; 5 mice/group; ^##^ *P* < 0.01 with respect to non-EAE control (CTR); *P < 0.05 and **P < 0.01 with respect to untreated EAE; scale bars, 100 μm

At pid-40, spinal cord myelin was evaluated using LFB staining and immunolabeling for MBP [23] (Fig. 2). Commensurate with the clinical findings, white matter tracts in untreated EAE mice stained weakly with LFB and immunolabeled weakly for MBP. Compared to normal controls (no EAE), the spinal cords of untreated EAE mice showed 37.9 ± 5.0% demyelination assessed by LFB, and 26.3 ± 3.4% by MBP. By comparison, the spinal cords of EAE/glib-pid-24 mice showed 23.5 ± 4.2% demyelination assessed by LFB, and 16.9 ± 1.9% by MBP.

Expression of the mature oligodendrocyte marker, 2′,3′-cyclic-nucleotide 3′-phosphodiesterase (CNPase), was used to verify the data on LFB and MBP (Fig. 2). Compared to normal controls, the spinal cords of untreated EAE mice showed 60.8 ± 4.6% demyelination, compared to 8.2 ± 2.1% in EAE/glib-pid-24 mice.

We performed counts of cells positive for platelet-derived growth factor receptor-α (PDGFR-α), a marker of oligodendrocyte precursor cells (OPC) (Fig. 2). Cells with PDGFR-α were significantly increased in both untreated EAE and EAE/glib-pid-24 mice, consistent with increased proliferation of OPC.

To assess for axonal sparing, spinal cords were examined for the pan-axonal neurofilament marker, SMI 312 (Fig. 2). Quantification revealed axonal loss of 54.9 ± 4.7% in untreated EAE mice, compared to normal controls, versus losses of 12.3 ± 1.6% in EAE/glib-pid-24 mice.

### Chronic phase glibenclamide reduces the inflammatory burden

**Fig. 3:**
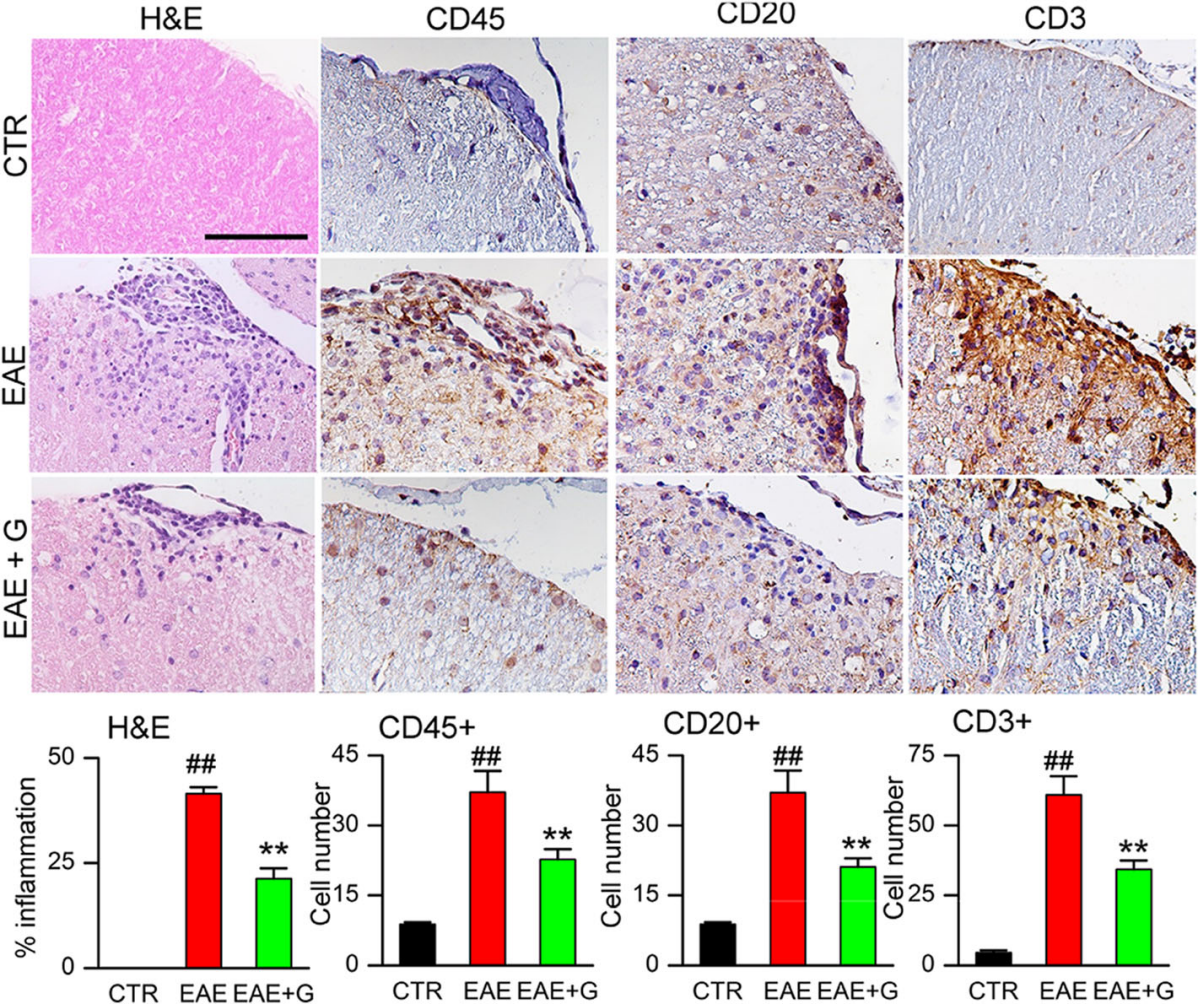
Chronic phase glibenclamide reduces the inflammatory burden in EAE. White matter of lumbar spinal cord sections from non-EAE control (CTR), untreated EAE mouse (EAE), and EAE mouse treated with glibenclamide starting on pid-24 (EAE + G), examined at pid-40, stained with H&E or immunolabeled for CD45 (leukocyte), CD20 (B cells), or CD3 (T cells), as indicated; original magnification, × 200 (H&E) or × 400 (all immunolabelings); *bar graphs:* percent of quadrants with inflammatory cells on H&E; quantification of CD45+, CD20+, and CD3+ cells in white matter; 5 mice/group; ^##^ *P* < 0.01 with respect to non-EAE control (CTR); **P < 0.01 with respect to untreated EAE; scale bars 100 μm

Lumbar spinal cords from normal controls (no EAE), untreated EAE mice, and EAE mice treated with glibenclamide beginning on pid-24 were examined by H&E staining (Fig. 3). Normal controls showed no inflammatory infiltrates, whereas meningeal, perivascular, and parenchymal inflammatory infiltrates were observed in the white matter of all untreated EAE spinal cords. Foci of inflammation were significantly reduced in the spinal cords of EAE/glib-pid-24 mice: 41.5 ± 1.5% of the quadrants in the untreated EAE mice were positive for inflammatory infiltrates, compared with 21.2 ± 2.4% of the quadrants in the EAE/glib-pid-24 group (Fig. 3).

To characterize the inflammatory cells present, sections of lumbar spinal cords were immunolabeled for markers of leukocytes (CD45), B cells (CD20), and T cells (CD3). Inflammatory lesions in untreated EAE lumbar spinal cords contained significantly increased numbers of CD45+, CD20+, and CD3+ cells, compared to normal controls (Fig. 3). The number of these cells was significantly reduced in EAE/glib-pid-24 mice (Fig. 3).

**Fig. 4:**
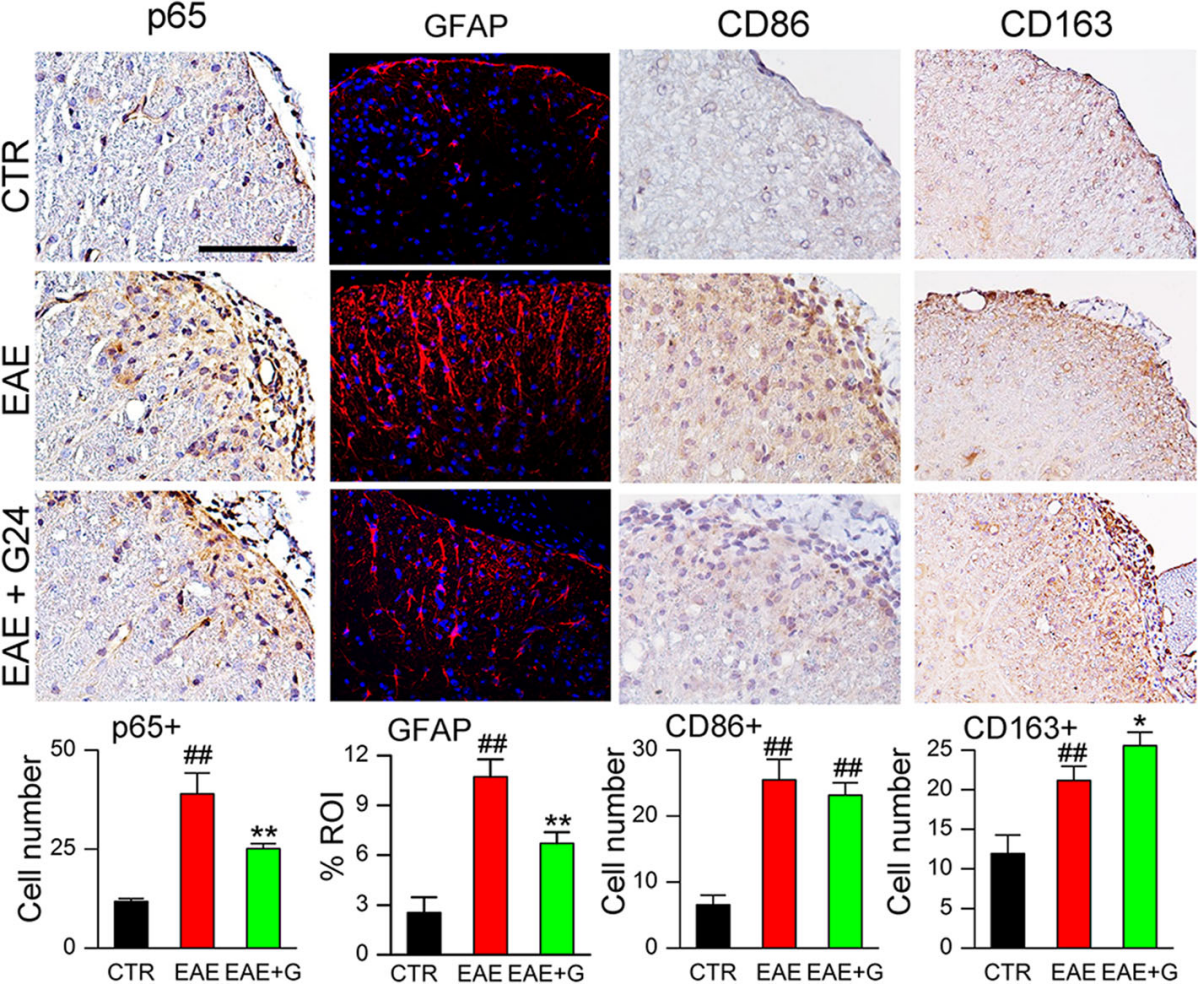
Chronic phase glibenclamide reduces inflammation and improves the macrophage phenotype in EAE. White matter of lumbar spinal cord sections from non-EAE control (CTR), untreated EAE mouse (EAE), and EAE mouse treated with glibenclamide starting on pid-24 (EAE + G), examined at pid-40, immunolabeled for p65 (NF-κB subunit), GFAP (astrocytosis), CD86 (M1 marker) or CD163 (M2 marker), as indicated; original magnification, × 400; *bar graphs:* quantification of p65+, GFAP, CD86+, and CD163+ cells in white matter; 5 mice/group; ^##^ *P* < 0.01 with respect to non-EAE control (CTR); *P < 0.05 and **P < 0.01 with respect to untreated EAE; scale bars 100 μm

To further characterize the effect of delayed glibenclamide administration on immune modulation in vivo, we counted cells positive for the nuclear factor-κB (NF-κB) subunit, p65. Cells positive for p65 were significantly increased in untreated EAE mice compared to normal controls (Fig. 4), whereas this increase was significantly blunted in EAE/glib-pid-24 mice (Fig. 4).

Similarly, reactive astrocytosis evaluated using GFAP immunoreactivity was markedly increased in untreated EAE mice compared to normal controls (Fig. 4), and this increase was significantly blunted in EAE/glib-pid-24 mice (Fig. 4).

We examined macrophage/microglial polarization using the M1 marker, CD86 [24], and the M2 marker, CD163 [24] (Fig. 4). Cells with prominent CD86 were significantly increased in untreated EAE mice compared to normal controls, with a similar increase in EAE/glib-pid-24 mice (Fig. 4). CD163 also was increased in untreated EAE mice, but was increased to a greater extent in EAE/glib-pid-24 mice (Fig. 4).

### Lymphocyte sequestration

**Fig. 5:**
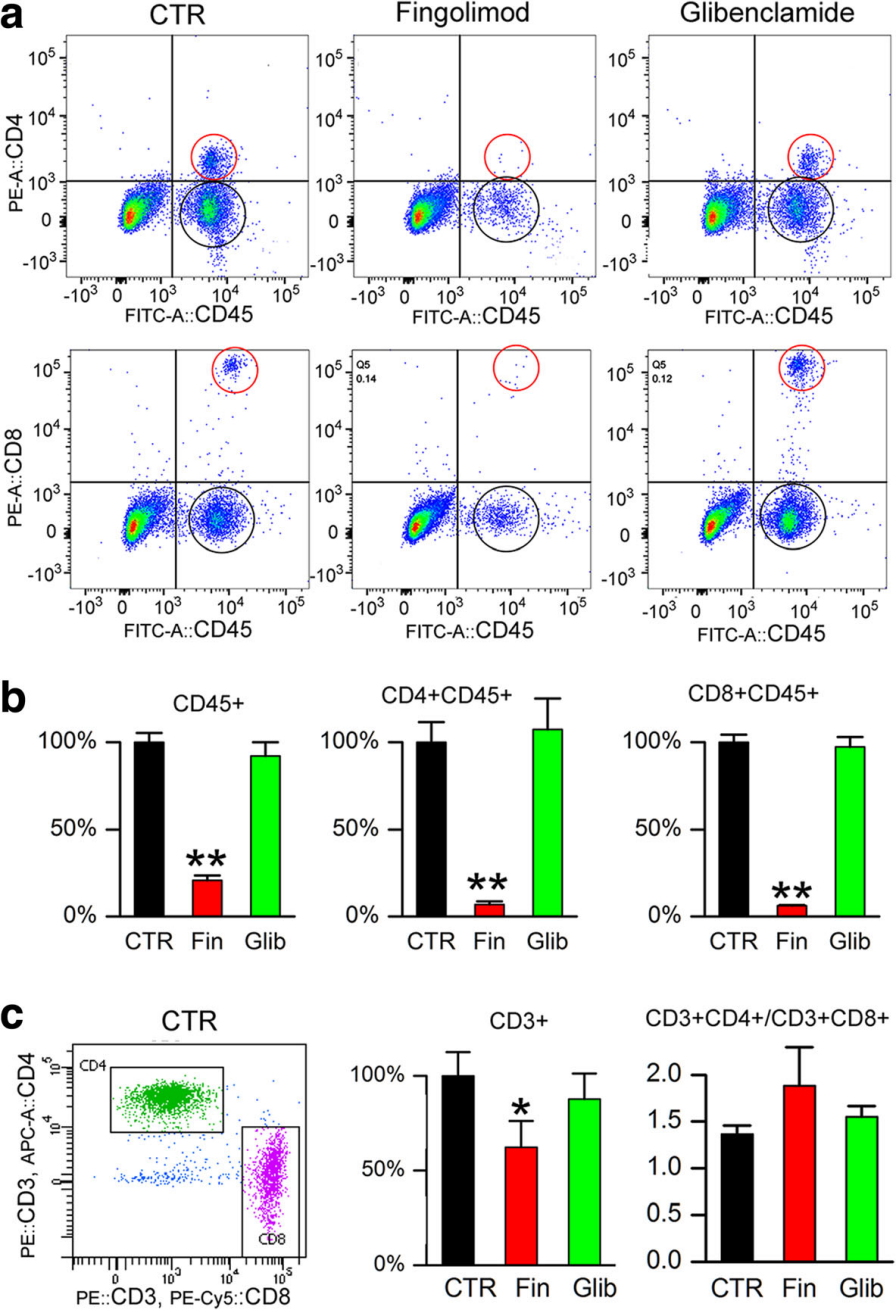
Glibenclamide does not alter circulating or splenic immune cell counts. Leukocyte suspensions were prepared from the blood or spleen of control mice or mice after 3 days of treatment with fingolimod (Fin; 3 mg/kg by gavage daily) or glibenclamide (Glib; 10 μg/mouse IP daily). **a**, **b** Representative plots (**a**) and quantification **b** showing the percent of total CD45+ leukocytes, and CD45 + CD4+ and CD45 + CD8+ T-lymphocytes in the three treatment groups, with respect to the control group, determined by FACS. **c** Representative plot and quantification showing the percent of total CD3+ cells with respect to the control group, and the ratios of CD3 + CD4+ to CD3 + CD8+ cells in the three treatment groups, determined by FACS; 3 mice/group; *P < 0.05 and **P < 0.01 with respect to control (CTR)

One possible explanation for the reduced inflammatory burden with glibenclamide could be that the drug acts to reduce the number of circulating peripheral immune cells, as is found with fingolimod, which acts in part by preventing lymphocyte egress from lymph nodes [25, 26]. We tested this hypothesis by treating normal (non-EAE) mice with either fingolimod or glibenclamide for 3 days, following which we used FACS to examine peripheral blood for two major classes of effector T cells, CD45 + CD4+ (helper), and CD45 + CD8+ (cytotoxic) cells. As expected, total CD45+ leukocytes as well as CD45 + CD4+ and CD45 + CD8+ T-lymphocytes were sharply reduced with fingolimod [19, 27] (Fig. 5a, b). By contrast, no changes in these cell counts were observed with glibenclamide treatment (Fig. 5a, b).

The distribution of lymphocyte subsets in the spleen is different from that in peripheral blood, with more CD8+ cytotoxic T cells found in the spleen [28]. We examined the effect of fingolimod and glibenclamide on the two major classes of T cells in the spleen, CD3 + CD4+, and CD3 + CD8+ cells. As with peripheral blood, reductions in cell numbers were observed with fingolimod [25], but not with glibenclamide, although the ratios of CD3 + CD4+ to CD3 + CD8+ cells [29] remained similar in the three groups (Fig. 5c).

### TNF, BAFF, CCL2, and NOS2 in EAE

**Fig. 6:**
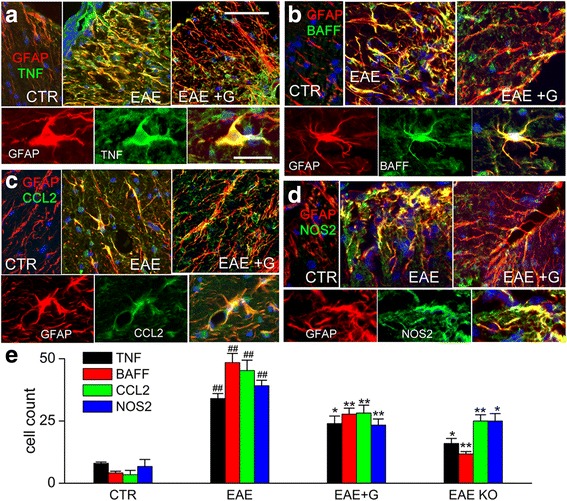
Chronic phase glibenclamide reduces TNF, BAFF, CCL2, and NOS2 in EAE. **a–d** Immunolabelings showing white matter of lumbar spinal cord sections from non-EAE control (CTR), untreated EAE mouse (EAE), and EAE mouse treated with glibenclamide starting on pid-24 (EAE + G), examined at pid-40, double immunolabeled for GFAP (red) and either TNF (**a**), BAFF (**b**), CCL2 (**c**) or NOS2 (**d**) (green); for each molecule, the upper panels show merged images of the color channels at low magnification, and the lower panels show, for the EAE condition, individual color channels and the merged images at high magnification. **e** Quantification of chromagen immunolabelings for TNF, BAFF, CCL2, and NOS2 in non-EAE controls (CTR), untreated EAE mice (EAE), EAE mice treated with glibenclamide starting on pid-24 (EAE + G), and in global *Abcc8−/−* mice with induced EAE; original magnification, × 400; 5 mice/group; ^##^ *P* < 0.01 with respect to non-EAE control (CTR); *P < 0.05 and **P < 0.01 with respect to untreated EAE; scale bars 100 and 25 μm, for upper and lower panels, respectively

Since SUR1-TRPM4 is expressed predominantly by astrocytes during the chronic phase of EAE [13], another possible explanation for the reduced inflammatory burden with glibenclamide could be that the drug acts on reactive astrocytes to reduce their expression of pro-inflammatory cytokines, chemokines, and neurotoxic factors. Tissues from lumbar spinal cords were double immunolabeled for GFAP and the cytokine, TNF [1, 30, 31], the B-cell immunostimulant, BAFF [9, 32–34], the leukocyte chemokine, CCL2 [35–37], or for a principal source of nitric oxide, NOS2 [8, 38, 39], all of which have been implicated in astrocytes in EAE or MS. In each case, double labeling of tissues from untreated EAE mice showed prominent expression of the four pro-inflammatory agents in astrocytes (Fig. 6a–e). By comparison, tissues from EAE/glib-pid-24 mice showed significantly less expression of the four pro-inflammatory agents by astrocytes (Fig. 6a–e).

We also examined the expression of TNF, BAFF, CCL2, and NOS2 in sections from the lumbar spinal cords of *Abcc8*−/− mice subjected to EAE; the favorable clinical course of these animals was reported previously [13]. Similar to findings with glibenclamide, gene suppression of *Abcc8* also was associated with significantly less TNF, BAFF, CCL2, and NOS2, compared to untreated WT EAE mice (Fig. 6e). These finding are consistent with glibenclamide acting via SUR1, not via an off-target mechanism.

### Glibenclamide tissue penetration

The foregoing findings suggested that glibenclamide was acting centrally. Normally, however, glibenclamide exhibits poor CNS penetration [40]. In EAE lesions, blood-brain and blood-spinal cord barriers are known to be compromised, as evidenced by extravasation of serum proteins such as albumin [41, 42]. Since glibenclamide in the circulation is highly protein bound (> 99%) [43, 44], we reasoned that extravasated serum proteins would transport glibenclamide into EAE lesions. To test this, we administered the fluorescent derivative of glibenclamide, bodipy-glibenclamide, to mice with clinically symptomatic EAE, and we assessed lumbar spinal cord lesions for albumin and for the fluorescent bodipy label.

**Fig. 7:**
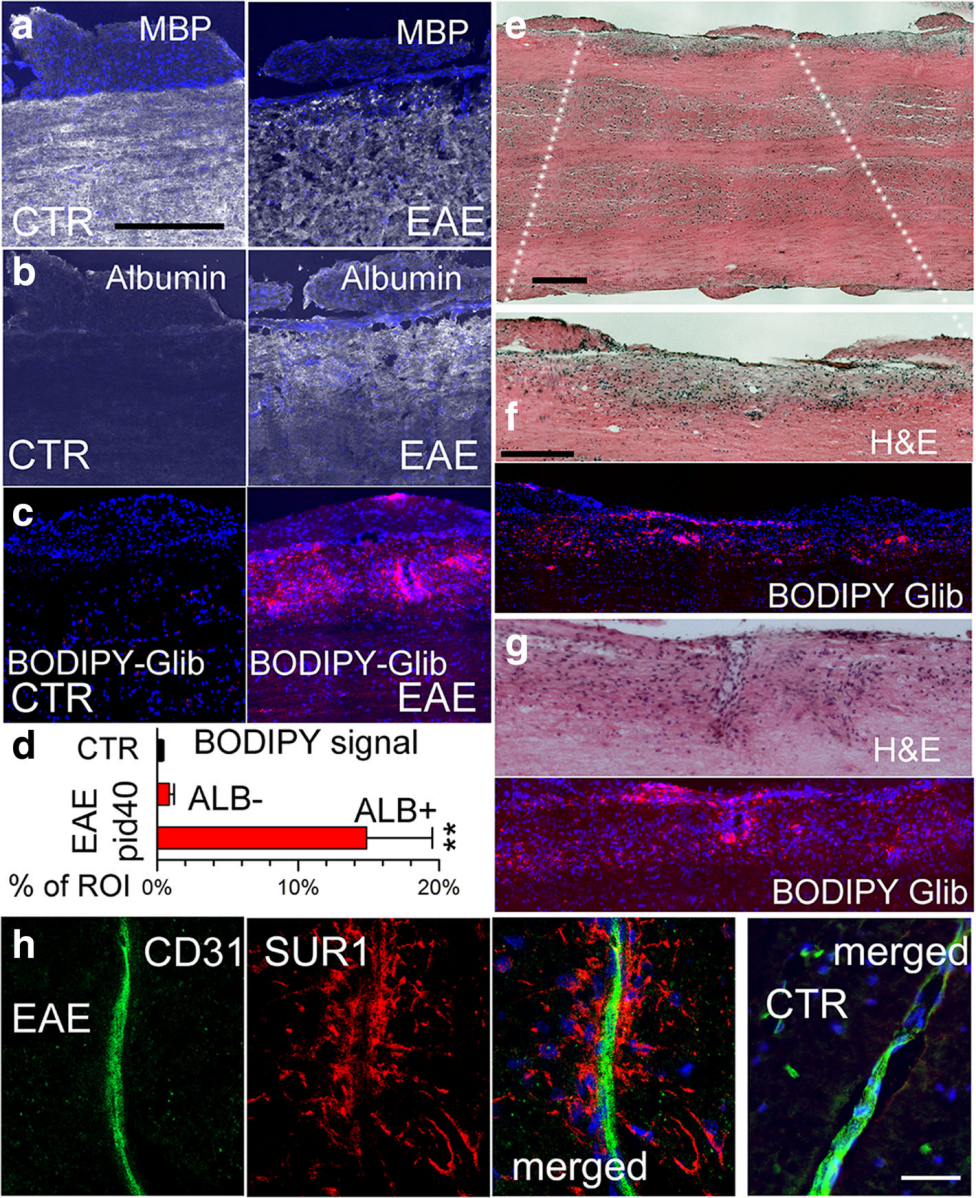
Glibenclamide enters EAE lesions in the spinal cord. **a**–**c** Sections of lumbar spinal cords from control (CTR) and EAE mice administered bodipy-glibenclamide, immunolabeled for MBP (**a**) or albumin (**b**), using an Alexa Fluor 488-conjugated secondary antibody (green signal, with signal shown as white for clarity), or imaged for intrinsic fluorescence due to bodipy (red) (**c**); nuclei labeled with DAPI (blue); scale bar 100 μm. **d** Quantification of bodipy signal in control (non-EAE) and in albumin-negative (ALB–) and albumin-positive (ALB+) regions of interest (ROI) from EAE; **P < 0.01 with respect to control (CTR). **e**–**g** Sections of lumbar spinal cords from EAE mice administered bodipy-glibenclamide, stained with H&E, illustrating a demyelinating lesion at low (**e**) and higher **f** magnification, and a non-demyelinating lesion with immune cell infiltrates (**g**), along with the associated intrinsic fluorescence due to bodipy (red); scale bars 200 μm. **h** Double immunolabeling of demyelinating lesion (*3 left panels*) and control tissue (*right panel*) for CD31 (endothelium) (green) and SUR1 (red) illustrates that SUR1 is not upregulated in endothelium but predominantly in perivascular structures typical of astrocytic endfeet; merged images are indicated; scale bars 25 μm

As expected [41, 42], immunolabeling showed abundant albumin extravasation within demyelinating lesions, compared to non-lesion tissues (Fig. 7a, b). In addition, demyelinating lesions showed prominent fluorescent labeling due to bodipy-glibenclamide, which was absent from non-lesion tissues in EAE mice as well as from control normal mice (Fig. 7c–f). In addition, non-demyelinating lesions, which were identified by immune cell infiltrates, also showed prominent fluorescent labeling due to bodipy-glibenclamide (Fig. 7g). These data indicate that glibenclamide can reach central targets when blood-brain and blood-spinal cord barriers are compromised by inflammation.

In CNS ischemia, SUR1 is highly expressed in microvascular endothelium, where it accounts for protein extravasation (vasogenic edema) [45]. This prompted us to question whether SUR1 expression in microvessels could similarly be linked to protein extravasation in EAE lesions. However, in contrast to ischemia, in EAE, SUR1 expression was found to be minimal or absent in microvascular endothelium, whereas it was very prominent in perivascular structures consistent with astrocytes and astrocytic foot processes (Fig. 7h).

### SUR1-TRPM4 and Tnf, Baff, Ccl2, and Nos2 mRNA in astrocytes in vitro

**Fig. 8:**
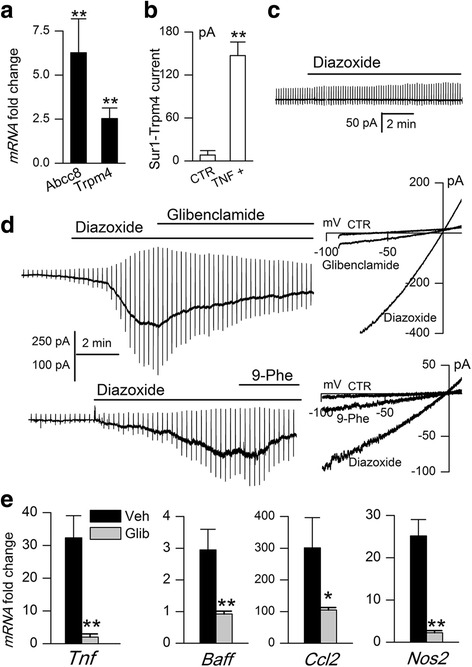
Inhibition of SUR1-TRPM4 channels with glibenclamide reduces *Tnf*, *Baff*, *Ccl2*, and *Nos2* mRNA in activated astrocytes. **a**–**d** Exposure of astrocytes to TNF + IFNγ (20 ng/mL each, overnight) upregulated mRNA for *Abcc8* and *Trpm4* (**a**), and induced the expression of functional SUR1-TRPM4 channels (**b**, **d**) that were not detected in non-activated astrocytes (**b**, **c**); note that SUR1-TRPM4 channel currents are activated by the SUR1-activator, diazoxide, and are blocked by the SUR1-inhibitor, glibenclamide, and the TRPM4 inhibitor, 9-phenanthrol (9-Phe) (**d**); *n* = 8–12 cells/condition. **e** Fold-increase in mRNA for *Tnf*, *Baff*, *Ccl2*, and *Nos2*, induced by activation of astrocytes by TNF + IFNγ, observed in the presence of vehicle (Veh) or glibenclamide (10 μM); *n* = 3–5 cultures/condition; *P < 0.05; **P < 0.01

The foregoing experiments suggested that SUR1-TRPM4 channels in astrocytes might be the target of glibenclamide and might be involved in the secretion of pro-inflammatory mediators by astrocytes. To explore this, we modeled the astrocyte response in MS/EAE by exposing primary astrocyte cultures to TNF plus IFNγ overnight [1]. Cell lysates analyzed by qPCR showed upregulation of mRNA for *Abcc8* and *Trpm4* (Fig. 8a), which encode the SUR1 and TRPM4 subunits, respectively.

Patch clamp electrophysiology was used to determine whether functional SUR1-TRPM4 channels were being upregulated by TNF + IFNγ-mediated astrocyte activation. For these experiments, intracellular and extracellular solutions contained Cs^+^ as the principal monovalent cation, because Cs^+^ blocks all K^+^ channels, including K_ATP_ channels, but it permeates non-selective cation channels such as SUR1-TRPM4. Channel currents were induced using diazoxide, which activates SUR1-regulated channels exclusively. Control non-activated astrocytes showed no response to diazoxide, indicating that quiescent astrocytes do not express SUR1-regulated cationic channels (Fig. 8b, c). By contrast, in activated astrocytes, diazoxide elicited large inward currents at physiological potentials that were blocked by the SUR1 or TRPM4 inhibitors, glibenclamide or 9-phenanthrol, respectively (Fig. 8b, d), consistent with the expression of functional SUR1-TRPM4 channels [12].

Exposure of astrocytes to TNF + IFNγ induced not only the expression of SUR1-TRPM4 channels but also upregulated mRNA for *Tnf*, *Baff*, *Ccl2*, and *Nos2*, with mRNA abundance being many-fold greater than in control astrocytes not activated by TNF + IFNγ [32] (Fig. 8e). Incubation with TNF + IFNγ in the presence of glibenclamide resulted in significant attenuation of mRNAs for *Tnf*, *Baff*, *Ccl2*, and *Nos2* (Fig. 8e), replicating in vitro some of our in vivo observations.

### SUR1-TRPM4 in demyelinating MS lesions

To explore the potential relevance of the aforementioned findings to MS, post-mortem tissues from the cerebral hemispheres of patients with MS and controls were assessed for SUR1-TRPM4 expression. Tissues were analyzed by histopathologic methods to identify areas of inflammation and demyelination. Demyelinating white matter lesions were categorized using CD68, CD45, and CD3 immunolabeling as per De Groot et al. [16], as active, chronic active, or chronic inactive. We also analyzed subpial/cortical areas of demyelination [46, 47].

**Fig. 9:**
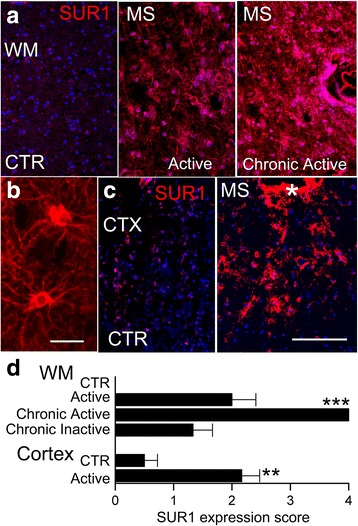
SUR1 is upregulated in demyelinating white matter and cortical lesions in MS patients. **a**, **b** Immunolabeling for SUR1 in control (CTR) white matter (WM) (**a**, *left*) and in demyelinating active (**a**, *middle*) and chronic active (**a**, *right*) WM lesions, shown at low (**a**) and high (**b**) magnification. **c** Immunolabeling for SUR1 in control (CTR) cortex (CTX) (**c**, *left*) and in demyelinating cortical lesion (**c**, *right*); asterisk, bottom of a sulcus; scale bars 250 μm (**a**, **c**) and 50 μm (**b**). **d** Semi-quantitative assessment of SUR1 immunolabeling in different types of demyelinating lesions; *n* = 4–6 lesions/type; note that CTR WM exhibited no specific labeling for SUR1; **P < 0.01, ***P < 0.001 with respect to respective controls (CTR)

We examined the expression of SUR1 in these various tissues. As illustrated in Fig. 9a, control white matter showed no specific labeling for SUR1, active white matter lesions showed distinct SUR1 labeling of stellate, astrocyte-like cells (Fig. 9b), and chronic active white matter lesions exhibited intense, widespread labeling of cells and processes. SUR1 immunolabeling also was prominent in subpial/cortical lesions, whereas control cortex showed only faint labeling in scattered large neuron-like cells (Fig. 9c). Semi-quantitative evaluation showed that different lesion types exhibited different levels of SUR1 expression, with chronic active lesions having the most intense labeling (Fig. 9d).

**Fig. 10:**
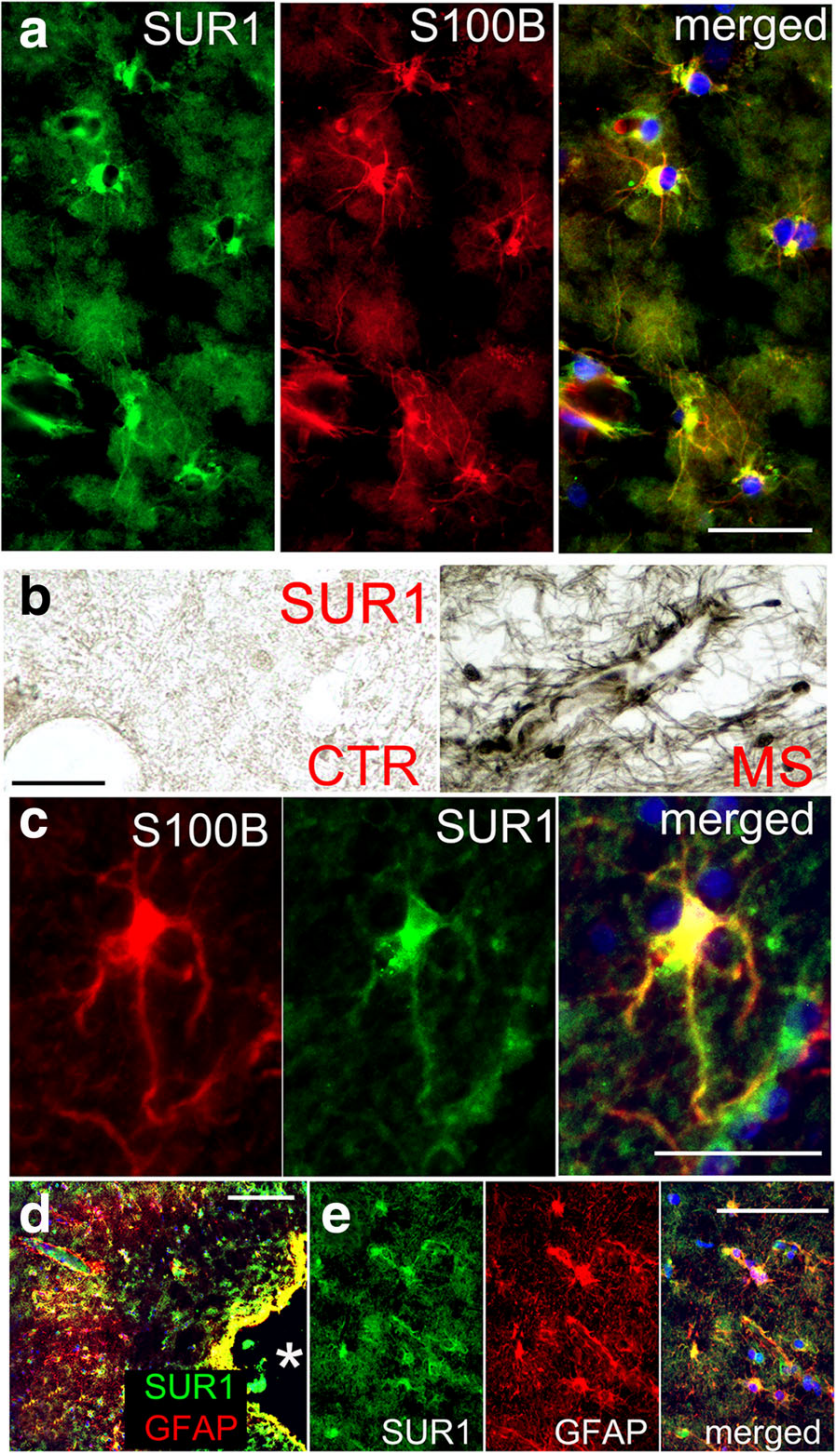
SUR1 is upregulated in astrocytes in demyelinating MS lesions. **a** Co-immunolabeling for SUR1 (green) and S100B (red) in a chronic active lesion; merged images are indicated; representative of 13 active, chronic active, or chronic inactive WM lesions. **b**, **c** Perivascular astrocytes, shown at low (**b**, *right*) and high (**c**) magnification, extend SUR1-positive processes toward nearby vessels, as visualized by chromagen (**b**) and fluorescent (**c**) immunolabeling; note absent SUR1 expression in control white matter (**b**, *left*). **d**, **e** Co-immunolabeling for GFAP (red) (**d**) and SUR1 (green) in a subpial/cortical demyelinating lesion, shown at low (**d**) and high (**e**) magnification; asterisk, bottom of a sulcus; representative of 6 cortical lesions; scale bars 50 μm (**a**, **c**) and 100 μm (**b**, **d**)

Double immunolabeling was carried out to identify the cell type (s) involved. Co-labeling for S100B showed that, in white matter lesions, SUR1 was expressed predominantly by astrocytes (Fig. 10a). Double labeling for SUR1 and CD68/ED1 (microglia/macrophage) or CD3 (T cells) showed minimal or no overlap with SUR1 expression (not shown). Stellate cells expressed SUR1 not only in the cell body but also in processes—in some chronic active white matter lesions, perivascular astrocytes could be seen to extend processes ending in perivascular end-feet that immunolabeled strongly for SUR1 (Fig. 10b, c). In subpial/cortical lesions as well, SUR1 expression was found predominantly in GFAP+ astrocytes, including the glia limitans (Fig. 10d, e).

**Fig. 11:**
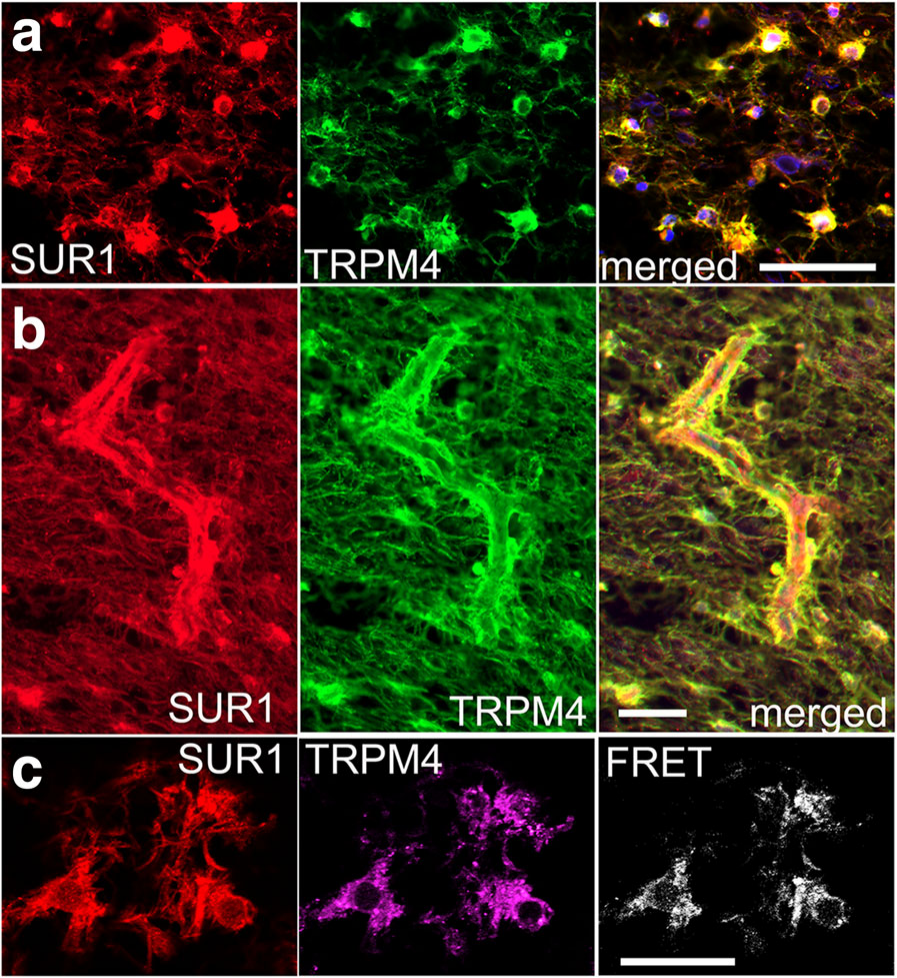
SUR1 and TRPM4 co-localize and co-assemble in demyelinating MS lesions. **a**, **b** Co-immunolabeling for SUR1 and TRPM4 in chronic active white matter lesions; merged images confirm co-localization of SUR1 and TRPM4 in white matter astrocytes (**a**) including perivascular astrocytes (**b**); representative of 13 active, chronic active, or chronic inactive lesions. **c** Co-immunolabeling for SUR1 and TRPM4 in chronic active white matter lesion, with immunoFRET confirming that SUR1 and TRPM4 co-assemble to form heteromers; representative of 13 active, chronic active, or chronic inactive WM lesions; scale bars 100 μm (**a**, **b**) and 25 μm (**c**)

Double immunolabeling showed co-expression of SUR1 and TRPM4 in stellate cells (Fig. 11a), as well as in perivascular end-feet (Fig. 11b). ImmunoFRET showed that SUR1 and TRPM4 physically co-assembled to form heterodimers (Fig. 11c), consistent with assembly of functional SUR1-TRPM4 channels [12].

**Fig. 12:**
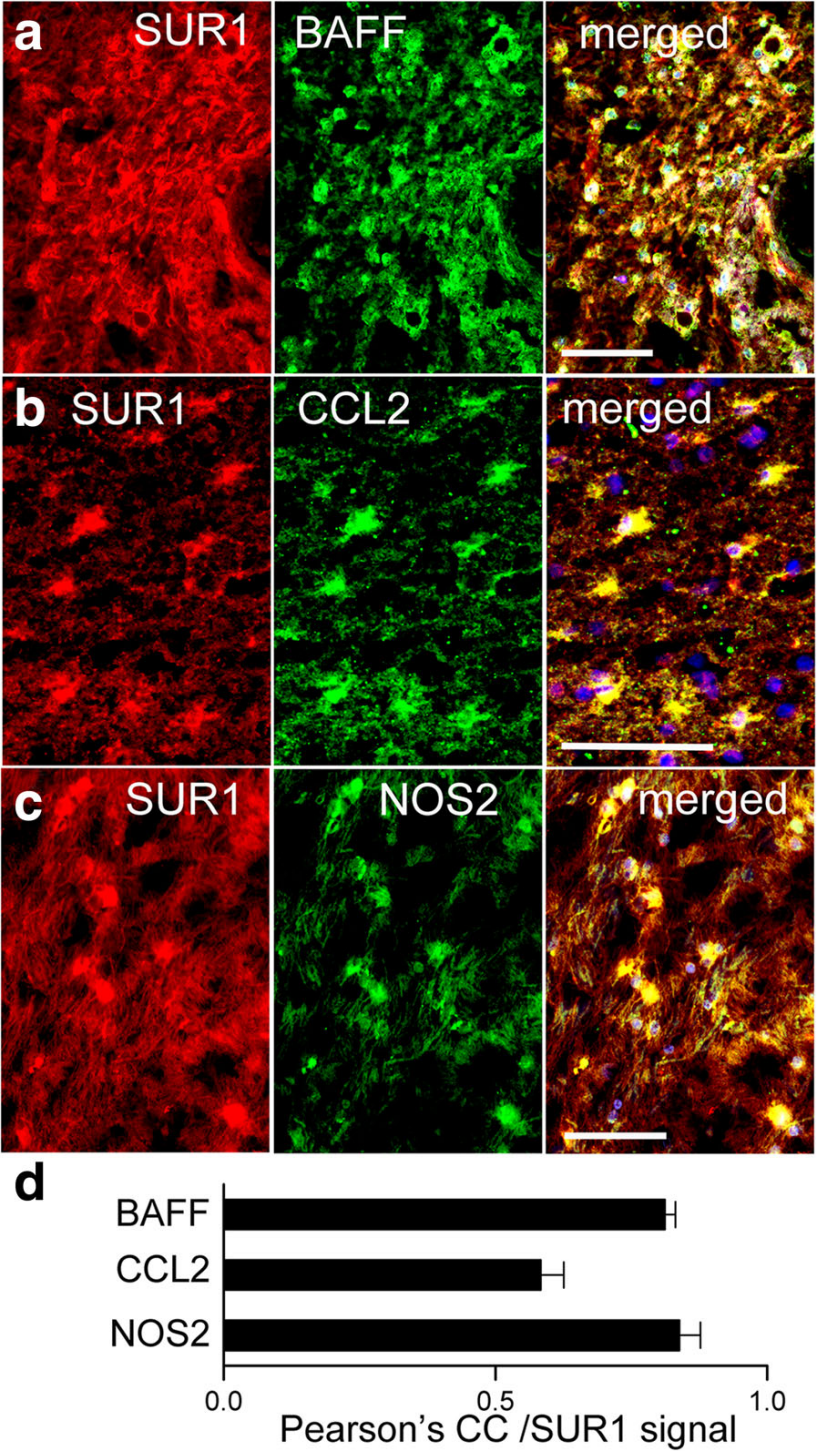
In demyelinating MS lesions, cells expressing SUR1 also express BAFF, CCL2, and NOS2. **a**–**c** Immunolabeling for SUR1 (red) and BAFF (**a**), CCL2 (**b**), or NOS2 (**c**) (green) in chronic active white matter lesions; merged images are indicated; scale bars 100 μm. **d** Quantitative assessment using Pearson’s correlation coefficient (CC) of SUR1 co-localization with BAFF, CCL2, or NOS2; *n* = 5–7 lesions per labeling

In MS white matter lesions, astrocytes were previously reported to upregulate BAFF [32], CCL2 [35], and NOS2 [38, 39]. Co-immunolabeling for SUR1 and BAFF, CCL2 or NOS2 confirmed that astrocytes in MS lesions that express SUR1-TRPM4 channels also express these pathogenic molecules (Fig. 12). High values of Pearson’s correlation coefficient for each one (0.58–0.84) reflect a high degree of co-localization with SUR1.

## Discussion

In a previous report [13], we showed that, in murine MOG_35–55_ EAE, global *Abcc8* deletion as well as SUR1 inhibition with glibenclamide starting on pid-10 reduced tissue leukocytes (CD45), T cells (CD3), B cells (CD20), and macrophages/microglia (CD11b), and reduced tissue pro-inflammatory cytokines (TNF, IFN-γ, IL-17). The reduced inflammatory burden with *Abcc8*/SUR1 deletion/inhibition was associated with better preservation of myelin (LFB and MBP), more numerous mature and precursor oligodendrocytes (CNPase, Olig2, PDGFR-α), better preservation of axons (SMI-312, AgNO_3_), and better neurological function. Notably, the therapeutic dose of glibenclamide needed for these effects is not sufficient to induce hypoglycemia [13].

Here, we expanded on those findings in three important, novel ways. Firstly, we showed that glibenclamide treatment could be started during the more clinically relevant, chronic phase of EAE, on pid-24, and still yield important therapeutic benefit. Glibenclamide started on pid-24 reduced the clinical severity, demyelination and the inflammatory burden, including tissue leukocytes (CD45), T cells (CD3), B cells (CD20), the p65 NF-κB subunit, and reactive astrocytosis, while promoting the macrophage M2 phenotype (CD163) over the M1 phenotype [48]. Secondly, we provide evidence that the mechanism of action of glibenclamide in reducing the inflammatory burden in EAE may be linked to a reduction in SUR1-mediated TNF, BAFF, CCL2, and NOS2 expression by astrocytes—the in vivo effects of drug on TNF, BAFF, CCL2, and NOS2 expression were replicated by global *Abcc8* deletion, consistent with glibenclamide acting via SUR1, and the in vivo effects of drug on TNF, BAFF, CCL2, and NOS2 expression were corroborated in vitro with primary astrocyte cultures. Thirdly, we provide evidence that astrocytic SUR1-TRPM4 channels are expressed not only in EAE but in MS as well, where they co-localize with BAFF, CCL2, and NOS2.

TRPM4 upregulation previously was identified in tissues from patients with MS [14]. Here, for the first time, we demonstrate co-expression of SUR1 along with TRPM4 in MS, and we present evidence based on immunoFRET that SUR1 and TRPM4 co-assemble in astrocytes of MS lesions to form SUR1-TRPM4 heteromers. In all types of demyelinating lesion examined, the expression of SUR1-TRPM4 was most prominent in astrocytes, with the greatest levels of expression found in chronic active lesions. The dominant expression of SUR1-TRPM4 in astrocytes, as found here in tissues from MS patients, accords with our previous observation during the chronic phase of murine EAE, when astrocytes are the predominant cells that express SUR1-TRPM4 [13].

The prominent expression of SUR1-TRPM4 in astrocytes is in keeping with the recognition of a critical role for astrocytes as immune effector cells with an essential role in MS and EAE [1, 2, 49, 50]. As active players in CNS innate immunity, astrocytes participate actively and differently at different stages of the pathologic process [2]. Astrocytes contribute mechanistically to lesion development in MS by (i) modifying blood-brain barrier properties and upregulating adhesion molecules and matrix metalloproteases required for leukocyte invasion; (ii) expressing cytokines and chemokines that attract leukocytes; (iii) producing factors toxic to oligodendrocytes and neurons; (iv) blocking the maturation of oligodendrocyte precursor cells; (v) forming a dense scar containing a small number of axons, glial cells, and little else. Activation of astrocytes in EAE occurs at the onset of the acute clinical episode, with the intensity being a good predictor of the clinical severity in animal models [51]. Astrocytes are the first cells in the CNS to be activated and to synthesize pro-inflammatory cytokines and chemokines that are essential for the induction of EAE [52].

Based on the foregoing, astrocytes may seem to be an attractive treatment target in MS/EAE. However, it is well recognized that simply eliminating astrocytes or broadly compromising their function worsens EAE severity [53, 54]. What is needed instead is a way to manipulate astrocyte function to obtain a more favorable phenotype. The present study, coupled with our previous work [13], shows that astrocytes are the predominant cell type that expresses SUR1-TRPM4, and that marked salutary effects are obtained in EAE by targeting SUR1 by (i) global gene deletion prior to EAE induction; (ii) pharmacological inhibition at the time of disease onset (pid-10 or pid-12); (iii) pharmacological inhibition during the chronic phase of EAE (pid-24). Together, these findings suggest that glibenclamide may be promising for disease modification in MS. Several disease-modifying therapies are available for MS, especially for relapsing-remitting forms of the disease. For the most part, these agents target a single immune mechanism, and they typically are used as sequential monotherapies, or as part of an escalation or induction strategy [5, 6]. Our findings suggest that targeting SUR1-TRPM4 in astrocytes could potentially yield the equivalent of a “combination therapy” by simultaneously reducing multiple pro-inflammatory and pathogenic mediators that contribute to disease progression.

Retrospective reviews of patients with diabetes mellitus type 2 who presented with an acute ischemic stroke revealed that they were less likely to have hemorrhagic transformation of their infarct and were more likely to have a better clinical outcome at discharge if they were on and were maintained on a sulfonylurea drug such as glibenclamide, compared to diabetics managed without sulfonylureas [55, 56]. The work presented here and previously [13] suggests that a similar analysis of diabetic patients with MS may be warranted.

This study has limitations. First, although astrocytes were found to be the dominant cell type expressing SUR1-TRPM4 in chronic EAE and MS, definitive characterization of the role of SUR1-TRPM4 in astrocytes will require the study of conditional knockout animals with astrocyte-specific deletion of *Abcc8* or *Trpm4*. Second, although we found that circulating leukocyte counts were not affected by glibenclamide administered to normal animals, these findings do not completely exclude a potential contribution of non-CNS effects of the drug in EAE. Third, although the blood-brain/blood-spinal cord barrier is known to be compromised in EAE [57], and here we showed that glibenclamide is transported across the compromised blood-spinal cord barrier, the mechanism by which systemically administered glibenclamide enters the CNS to affect disease progression has not been fully elucidated. Fourth, although beyond the scope of this study, determining the potential involvement of SUR1-TRPM4 in preactive white matter lesions with “normal appearing white matter” will be important to elucidate the role, if any, of SUR1-TRPM4 in MS pathogenesis, including the relation between astrocytic SUR1-TRPM4 and the microglial clusters that comprise the earliest identified abnormality in MS [58, 59].

## Conclusions

The SUR1-TRPM4 channel is upregulated in chronic phase murine EAE and human MS. Given its predominant expression in reactive astrocytes, and the important pro-inflammatory role of astrocytes in MS and EAE, the SUR1-TRPM4 channel may represent a novel therapeutic approach for disease modification to reduce peripheral immune cell entry into the CNS without compromising the function of peripheral immune cells.

## Abbreviations

BAFF: B-cell activating factor
CCL2: Chemokine (C-C motif) ligand 2
CD: Cluster of differentiation
CNPase: 2′,3′-cyclic-nucleotide 3′-phosphodiesterase
CNS: Central nervous system
DAPI: 4′,6-diamidino-2-phenylindole
DMSO: Dimethylsulfoxide
EAE: Experimental autoimmune encephalomyelitis
EAE/glib-pid-12: Glibenclamide treatment of EAE beginning on pid-12
EAE/glib-pid-24: Glibenclamide treatment of EAE beginning on pid-24
FACS: Fluorescence-activated cell sorting
GFAP: Glial fibrillary acidic protein
H&E: Hematoxylin and eosin
IFNγ: Interferon γ
immunoFRET: Antibody-based Förster resonance energy transfer
IP: Intraperitoneally
LFB: Luxol fast blue
MBP: Myelin basic protein
MOG: Myelin oligodendrocyte glycoprotein
MS: Multiple sclerosis
NF-κB: Nuclear factor-κB
NOS2: Nitric oxide synthase 2
NS: Normal saline
PBS: Phosphate buffered saline
PDGFR-α: Platelet-derived growth factor receptor α
Pid: Post-induction day
qPCR: Quantitative polymerase chain reaction
S1P: Sphingosine-1-phosphate
SE: Standard error of the mean
SUR1-TRPM4: Sulfonylurea receptor 1–transient receptor potential melastatin 4
TNF: Tumor necrosis factor
WT: Wild-type
